# Detailed spatial immunophenotyping of primary melanomas reveals immune cell subpopulations associated with patient outcome

**DOI:** 10.3389/fimmu.2022.979993

**Published:** 2022-08-08

**Authors:** Grace H. Attrill, Hansol Lee, Annie T. Tasker, Nurudeen A. Adegoke, Angela L. Ferguson, Ines Pires da Silva, Robyn P. M. Saw, John F. Thompson, Umaimainthan Palendira, Georgina V. Long, Peter M. Ferguson, Richard A. Scolyer, James S. Wilmott

**Affiliations:** ^1^ Melanoma Institute Australia, The University of Sydney, Sydney, NSW, Australia; ^2^ Faculty of Medicine and Health, The University of Sydney, Sydney, NSW, Australia; ^3^ Charles Perkins Centre, The University of Sydney, Sydney, NSW, Australia; ^4^ Centenary Institute, The University of Sydney, Sydney, NSW, Australia; ^5^ Westmead and Blacktown Hospitals, Sydney, NSW, Australia; ^6^ Royal Prince Alfred Hospital, Sydney, NSW, Australia; ^7^ Mater Hospital, North Sydney, NSW, Australia; ^8^ Royal North Shore Hospital, St Leonards, NSW, Australia; ^9^ NSW Health Pathology, Sydney, NSW, Australia

**Keywords:** primary melanoma, immunophenotyping, spatial pathology, T cell phenotypes, Clinicopathological features

## Abstract

While the tumor immune microenvironment (TIME) of metastatic melanoma has been well characterized, the primary melanoma TIME is comparatively poorly understood. Additionally, although the association of tumor-infiltrating lymphocytes with primary melanoma patient outcome has been known for decades, it is not considered in the current AJCC melanoma staging system. Detailed immune phenotyping of advanced melanoma has revealed multiple immune biomarkers, including the presence of CD8+ T-cells, for predicting response to immunotherapies. However, in primary melanomas, immune biomarkers are lacking and CD8+ T-cells have yet to be extensively characterized. As recent studies combining immune features and clinicopathologic characteristics have created more accurate predictive models, this study sought to characterize the TIME of primary melanomas and identify predictors of patient outcome. We first phenotyped CD8+ T cells in fresh stage II primary melanomas using flow cytometry (n = 6), identifying a CD39+ tumor-resident CD8+ T-cell subset enriched for PD-1 expression. We then performed Opal multiplex immunohistochemistry and quantitative pathology-based immune profiling of CD8+ T-cell subsets, along with B cells, NK cells, Langerhans cells and Class I MHC expression in stage II primary melanoma specimens from patients with long-term follow-up (n = 66), comparing patients based on their recurrence status at 5 years after primary diagnosis. A CD39+CD103+PD-1- CD8+ T-cell population (P2) comprised a significantly higher proportion of intratumoral and stromal CD8+ T-cells in patients with recurrence-free survival (RFS) ≥5 years *vs* those with RFS <5 years (p = 0.013). Similarly, intratumoral B cells (p = 0.044) and a significantly higher B cell density at the tumor/stromal interface were associated with RFS. Both P2 and B cells localized in significantly closer proximity to melanoma cells in patients who remained recurrence-free (P2 p = 0.0139, B cell p = 0.0049). Our results highlight how characterizing the TIME in primary melanomas may provide new insights into how the complex interplay of the immune system and tumor can modify the disease outcomes. Furthermore, in the context of current clinical trials of adjuvant anti-PD-1 therapies in high-risk stage II primary melanoma, assessment of B cells and P2 could identify patients at risk of recurrence and aid in long-term treatment decisions at the point of primary melanoma diagnosis.

## Introduction

While melanoma is highly immunogenic (1, 2) and immune checkpoint inhibitors have greatly improved survival of advanced-stage melanoma patients, the tumor immune microenvironment remains poorly characterized in primary melanoma (3). Furthermore, patients with stage IIB/C primary melanoma have a higher risk of melanoma-related death compared to patients with stage IIIA/B metastatic melanomas (87-82% *vs* 93-83% 5-year survival) (4). While surgery is curative for the majority of primary melanoma patients, a recent clinical trial demonstrated improved recurrence-free survival in high-risk stage II (stage IIB/C) melanoma patients receiving adjuvant anti-PD-1 therapy (5). However, a proportion of patients experienced immune-related adverse events, some irreversible. Therefore, it is critical to identify high-risk primary melanoma patients and to provide them with the correct therapies.

The predictive capacity of many clinicopathologic features of primary melanoma have been well documented (6). Foremost among these are Breslow thickness and ulceration. Both of these features are included in the AJCC 8^th^ edition staging guidelines for melanoma (4), with both increased Breslow thickness and the presence of ulceration being poor prognostic indicators. The presence and distribution of tumor-infiltrating lymphocytes (TILs) as determined by pathological assessment is also closely associated with outcome (7, 8). However, the prognostic capacity of these characteristics can be closely entwined. For example, TILs are more predictive of outcome in thicker melanomas than they are in thin melanomas (9), and ulceration is also known to invoke an inflammatory immune response (4). Recent studies have addressed this by combining multiple clinicopathologic features (10), finding that TILs quantified *via* molecular means (T-cell fraction, TCRseq) provided superior prognostic capability when combined with Breslow thickness over all other established clinicopathologic variables (11).

In light of this, thorough characterization of the tumor immune microenvironment in primary melanoma is becoming increasingly relevant. CD8+ T cells form the majority of TILs and are the primary target of many immune checkpoint inhibitors. Furthermore, specific CD8+ T cell populations can act as biomarkers of response to immunotherapy in advanced stages (12, 13), and studies in primary melanoma have shown that CD8+ T cells may also predict recurrence following primary melanoma resection. In particular, CD8+ T cells expressing CD39, CD103 and PD-1 have been of great interest due to their potential functional roles. CD39 has recently been proposed as a marker of tumor antigen specificity (14, 15), CD103 is a marker of tumor resident memory CD8+ T cells (13, 16) and PD-1 is an immune checkpoint expressed by antigen-experienced CD8+ T cells (17, 18). CD69, when expressed independently from CD103, is also a marker of intermediate tumor residency (13, 16). Recent efforts to assess immune infiltrates in primary melanomas in a broader context have focused on gene expression profiling and imaging mass cytometry (19–23). Increasingly, spatial analysis has provided further insights into tumor and immune cell interactions in the TME (24–26).

In this study, we sought to identify immune cell features of the TME of primary melanomas prognostic of patient outcome. We performed spatial characterization of the primary melanoma immune microenvironment, specifically targeting CD8+ T cells, B cells, NK cells, Langerhans cells and Class I MHC expression using Opal multiplex immunohistochemistry (mIHC), and correlating these features with tumor clinicopathologic features and patient outcome data.

## Materials and methods

### Cohort and study design

All tissue was acquired for research with approval from the Sydney Local Health District Human Ethics Review Committee (protocol no X15-0454 and 2019/ETH06874), and informed consent from each patient and from the MIA Biospecimen Tissue Bank. Prospectively collected information from the Melanoma Institute Australia database was searched to identify patients diagnosed with primary melanomas >1mm Breslow thickness between 1999 and 2011 who had no systemic therapies. Stage at melanoma diagnosis was collected, and patients diagnosed with stage III *via* a positive sentinel node were included. Patients were grouped into ‘Good’ or ‘Poor’ based on their outcome at 5 years following diagnosis. Patients with recurrence-free survival of greater than 5 years since resection of the primary melanoma were classified as ‘Good’, while patients who died due to melanoma within 5 years of their primary excision were classified as ‘Poor’. Patients whose 5-year outcome was unknown, or did not fit either definition - such as those who recurred within 5 years but did not die, or patients who died of causes other than melanoma within 5 years - were excluded from the study. Patients were subdivided based on Breslow thickness (<2mm, 2-4mm, >4mm) and selected so that clinicopathologic characteristics, specifically Breslow thickness, ulceration status, anatomic site (head and neck *vs* other), age at diagnosis and gender, were matched between the subdivisions of each outcome group as closely as possible. Hematoxylin and eosin-stained slides were reviewed by a clinical pathologist (PMF) to confirm the primary melanoma diagnosis, assess tissue suitability for mIHC staining and obtain any missing pathologic data. Formalin-fixed paraffin-embedded (FFPE) primary melanoma tissue was collected from the Royal Prince Alfred Hospital and Douglass Hanly Moir Pathology for the selected patients for fluorescent multiplex immunohistochemistry analysis (n = 66, **Table 1**).

**Table 1:** Clinicopathologic characteristics of primary melanoma samples stained for IHC.

	Outcome	<2mm	2-4mm	>4mm	Total
**# of patients**		6	34	26	66
	Poor	3	17	13	33
	Good	3	17	13	33
**Breslow thickness (mm), median**	Poor	1.5	2.8	6.2	3.5
	Good	1.48	2.7	6	3.5
	**Total**	**1.5**	**2.75**	**6.1**	**3.5**
**Age at diagnosis (years), median**	Poor	47.83	66.56	68	66
	Good	63	59	65	64
	**Total**	**54.42**	**64.09**	**67.22**	**64**
**Gender (#F, #M)**	Poor	2F, 1M	5F, 12M	3F, 10M	10F, 23M
	Good	2F, 1M	7F, 10M	4F, 8M	13F, 20M
	**Total**	**4F, 2M**	**12F, 22M**	**7F, 18M**	**23F, 46M**
**Site (#H, #O)** **H = head, O = other**	Poor	1H, 2O	7H, 10O	3H, 10O	11H, 22O
	Good	1H, 2O	6H, 11O	3H, 10O	10H, 23O
	**Total**	**2H, 4O**	**13H, 21O**	**6H, 20O**	**21H, 45O**
**Ulceration (% ulcerated)**	Poor	1/3 (33.33%)	6/17 (35.29%)	9/13 (69.23%)	16/33 (48.48%)
	Good	1/3 (33.33%)	6/17 (35.29%)	9/13(69.23%), 1 unknown	16/33 (48.48%)
	**Total**	**2/6 (33.33%)**	**12/34 (35.29%)**	**18/276(69.23%)**	**32/66 (48.48%)**
**Stage at diagnosis (II, III)** **II = stage IIA, IIB, IIC** **III = stage IIIA, IIIB, IIIC**	Poor	1 II, 2 III	9 II, 8 III	4 II, 9 III	**14 II, 19 III**
	Good	1 II, 2 III	13 II, 4 III	11 II, 2 III	**25 II, 8 III**
	**Total**	**2 II, 4 III**	**22 II, 12 III**	**15 II, 11 III**	**39 II,** **27 III**

Bold values indicate values for total patients.

A separate retrospective validation cohort consisting of two tissue microarrays of 1mm^2^ tissue cores containing 122 primary melanoma FFPE samples from 81 patients with available follow-up details were collected regardless of stage of at melanoma diagnosis (stage I-IV). When tumor size permitted, multiple tissue cores were taken from the primary melanoma samples, and data was averaged between the two cores to create a single result for each patient in these cases. Patients within this cohort were diagnosed with primary melanoma from 2008-2013, and samples were collected from the Royal Prince Alfred Hospital. No matching of clinicopathologic data was performed for this cohort, and no patients received systemic therapies as of last follow-up.

Separately, fresh primary melanoma biopsies were collected in a prospective manner from patients who underwent surgical resection at Melanoma Institute Australia between January and October 2018. Fresh tissues were collected from the Royal Prince Alfred Hospital and the Mater Hospital. Tumor dissociates were generated from the melanoma biopsies using a Miltenyi Tumor Dissociation Kit and gentleMACS Octo dissociator (Miltenyi Biotec), then cryopreserved at -80°C for subsequent flow cytometry analysis. Nine suitable primary melanoma biopsies were available for flow cytometry staining. Investigators were blinded to patient outcome throughout the staining and analysis process for both flow cytometry and mIHC.

### Flow cytometry

Unstained and single-color controls were prepared alongside samples. Single-color controls were prepared by staining UltraComp eBeads (eBiosciences) with single fluorescent mAbs diluted in FACS buffer.

Up to 1 x 10^6^ cells per cryopreserved tumor sample were resuspended in Zombie Aqua™ diluted in PBS (1:100, Biolegend), then incubated at 4°C in the dark for 20 minutes. Cells were washed in PBS then stained with the following antibodies to identify T cell subsets: CD3 (BD Biosciences, SK7, 1:20), CD8a (BD Biosciences, SK1, 1:20), PD-1 (BD Biosciences, EH12.1, 1:20), CD39 (BD Biosciences, TU66, 1:50), CD69 (Biolegend, FN50, 1:50), VISTA (R&D Systems, 73084, 1:20), GITR (Biolegend, 108-17, 1:20), CD103 (Biolegend, BerACT8, 1:20), and CD4 (BD Biosciences, RPA-T4, 1:50). Following the addition of antibody, cells were incubated in the dark at 4°C for 20 minutes. Cells were washed in FACS buffer and resuspended in 10% PFA, then incubated for 30 minutes at 4°C. Cells were washed and resuspended in FACS buffer before flow cytometry analysis on a BD LSR-II flow cytometer (BD Biosciences). Flow cytometry data was processed using FlowJo v 10.2 (Tree Star) (**Supplementary Figure 1**).

### Multiplex immunohistochemistry

FFPE tissue was cut into 3µm thick sections and mounted on Superfrost Plus slides (ThermoFisher Scientific). Slides were air-dried overnight, then placed in a vacuum sealed dehydrator for short-term storage. mIHC panels were optimized as according to Yaseen et al. (27).

Following deparaffinization in xylene and gradual rehydration in decreasing concentrations of ethanol, slides were placed in AR 9 or AR 6 buffer (Akoya Biosciences) and heated to 110°C in a pressurized Decloaking Chamber (Biocare Medical) for 20 minutes, then cooled to room temperature in a water bath. All staining was performed using an intelliPATH FLX^®^ Automated Slide Stainer (Biocare Medical). Slides were first incubated with 3% H_2_O_2_ for 10 minutes to block endogenous peroxidase activity. Following a TBST wash, slides were incubated in sequential rounds of primary antibody for 30 minutes for either PD-1 (Abcam, EPR4877(2), 1:1500), CD103 (Abcam, ERP4166(2), 1:1500), CD8 (Dako, C8/144B, 1:1500), CD3 (Cell Marque, MRQ-39, 1:1500), or CD39 (Abcam, ERP20627, 1:2000) for the T cell panel; Langerin (Cell Marque, 12D6, 1:400), CD56 (Cell Marque, MRQ-42, 1:500), CD1a (Cell Marque, EP80, 1:4000), CD20 (Biocare, L26, 1:800), or HLA-ABC (Abcam, EMR8-5, 1:20000) for the immune panel; or SOX10 (Biocare Medical, BC34, 1:200) for both. Both cohorts were stained with the T cell panel (**Supplementary Table 1**), while the discovery cohort was also stained with the immune panel (**Supplementary Table 2**). Slides were stained with Mach 3 Rabbit probe or Mouse probe (Biocare Medical) for 10 minutes followed by Mach 3 Rabbit HRP or Mouse HRP for 10 minutes, or Opal Polymer HRP Ms + Rb (Akoya Biosciences) for 30 minutes. Following this, slides were incubated in Opal fluorophore (1:100, Akoya Biosciences) diluted in 1X Plus Amplification Diluent (Akoya Biosciences) for 10 minutes. After each Opal stain, slides were stripped of antibody *via* antigen retrieval as above to prepare for staining with the next antibody in the multiplex. Following the addition of Opal for the final antibody, slides were incubated with Spectral DAPI (1:2000, Akoya Biosciences) diluted in TBST for 5 minutes. Slides were mounted in Prolong Diamond Antifade Mountant (Thermo Fisher Scientific) and allowed to cure at room temperature overnight before imaging. Single color control, multiplex control and unstained control slides were stained alongside patient samples to determine background staining and create a spectral library for imaging and spectral unmixing.

### Multispectral imaging

Imaging of tissue sections was performed using a Vectra 3.0.5 Automated Quantitative Pathology Imaging system (Akoya Biosciences). 20X resolution (0.5µm/pixel) images encompassing the entire primary tumor and surrounding epidermis were acquired using the DAPI, FITC, Cy3, Texas Red and Cy5 channels. InForm v2.4.2 (Akoya Biosciences) was used for spectral unmixing based on a library created from single color controls.

### Image analysis

Primary image analysis was performed using HALO v3.0.1 (Indica Labs). A single high-resolution image of each patient’s tumor was created by stitching together individual 20X multispectral images. Tissue classification was used to stratify the tumor and surrounding epidermis and stroma. Cell segmentation was performed using an algorithm created from the intensity of nuclear DAPI or SOX10 staining. Positivity thresholds were set and reviewed individually for each sample based on cytoplasmic or nuclear staining intensity for each marker. Cells were phenotyped in HALO based on marker expression, and CD8+ T cells were phenotyped based on the expression of CD39, CD103 and PD-1, dividing them into 8 phenotypically distinct populations named P1 to P8, as reported previously (28). Data for each cell’s marker expression and X, Y location were stored in HALO. Cell counts for each phenotype were exported from HALO for statistical analysis.

Image registration was used to align images of serial sections stained with different panels. This allowed for plotting of phenotypes from both panels on the same plot, and for analysis of spatial relationships between cells from different panels. X, Y coordinates of cells from registered images were stored in the HALO software.

### Spatial analysis

Proximity, marginal and infiltration analysis were performed within the HALO Spatial Analysis module (Indica Labs). All phenotyped cells from both panels were registered and plotted together on a single plot. The Proximity Analysis tool was used to identify the average distance of each phenotype from melanoma cells and the percentage of each phenotype within 20 µm of a melanoma cell. The 20 µm cutoff was selected based on analysis from previous studies (25). As melanoma cells were stained separately on both panels, this analysis was done separately for each panel. The Infiltration Analysis tool was used to investigate immune populations at the tumor margin. Margins were drawn at the border between tumor and epidermis/stroma as determined by the tissue classifier and manually edited for improved accuracy. A band of 400µm width around the margin was analyzed, divided into 50µm-wide sections extending into the stroma (0 to 50µm, 50 to 100µm, 100 to 150 µm, 100 to 200µm) and tumor (0 to -50µm, -50 to -100µm, -100 to -150µm, -150 to -200µm). Immune cells were quantified separately within each band. Spatial data for all analyses in all phenotypes were exported for statistical analysis.

### Statistical analysis

Statistical analysis of immune cell quantities, spatial distribution and flow cytometry data was performed using an unpaired non-parametric Mann-Whitney test in GraphPad Prism v8. Correlation analysis and clustering was performed using the R corrplot package (version 0.84). Margin analysis was performed using a 2-way ANOVA or Kruskal-Wallis unpaired non-parametric test as required.

Clinicopathologic features and CD8+ T cell populations were analyzed using a univariable Cox proportional hazard ratio (**Table 2**). Random survival forest models with 10-fold cross-validation were fitted using the Python scikit-survival toolkit (29). These models used exhaustive feature selection testing all possible combinations of CD8+ T cell populations and clinicopathologic features in combinations of up to 6 variables at a time to determine the best predictive model. Kaplan-Meier curves were generated using the R survminer package (version 0.4.9). Cutoffs were determined by ROC curve analysis which maximized the sensitivity and specificity of the analysis and compared using a log-rank test. Peak AUCs are reported where relevant. All statistical analysis was performed in R and Python. P-values of <0.05 were considered statistically significant for all analyses.

**Table 2:** Univariate analysis of patient clinicopathologic characteristics and immune features in the discovery cohort.

Characteristics	Good (n = 29)	Poor (n = 25)	*p value*
**Age**			
**Median (Range)**	65 (31-86)	69 (47-92)	**0.04789**
**Gender**			
**Female**	11 (37.9%)	8 (32.0%)	0.8655
**Male**	18 (62.1%)	17 (68.0%)	
**Stage**			
**II (IIA/B/C)** **III (IIIA/B/C)**	21 (72.4%) 8 (27.6%)	12 (48.0%) 13 (52.0%)	0.1199
**Ulceration**			
**NO**	14 (48.3%)	10 (40.0%)	0.7371
**YES**	15 (51.70%)	15 (60.0%)	
**Nodal Status**			
**0**	21 (72.4%)	12 (48.0%)	0.1199
**1**	8 (27.6%)	13 (52.0%)	
**Breslow**			
**Median (Range)**	3.50 (1.35-9.0)	3.50 (1.5-8.0)	0.4929
**P1 mm2 tumor**			
**Median (Range)**	7.17 (0.0-981.26)	1.86 (0.0-288.29)	0.2115
**P2 mm2 tumor**			
**Median (Range)**	16.29 (0.68-622.31)	2.44 (0.14-175.48)	**0.01165**
**P4 mm2 tumor**			
**Median (Range)**	30.49 (2.50-696.15)	21.61 (1.68-634.24)	0.1510
**P6 mm2 tumor**			
**Median (Range)**	12.31(0.34-237.34)	3.20 (0.0- 111.85)	0.08277
**% P2 tumor**			
**Median (Range)**	10.50 (1.20-30.23)	5.09 (0.10-23.32)	**0.005152**
**% P2 stroma**			
**Median (Range)**	6.84 (0.81-15.49)	2.77 (0.52-22.31)	**0.006105**

Bold values indicate significant values.

## Results

### Patient characteristics

A cohort of 66 patients diagnosed with primary melanoma >1mm Breslow thickness from 1999-2011 was included in the study (**Table 1**). Resected primary melanoma FFPE tissue was collected from each patient. All patients underwent primary melanoma resection, and none were treated with any systemic drug therapies. Within this cohort, 33 patients (50%) died of metastatic melanoma within 5 years of primary melanoma diagnosis (Poor outcome) and 33 patients remained alive and recurrence-free following primary diagnosis, with at least 5 years of follow-up (Good outcome). The median time from diagnosis to death in the poor outcome group was 2.3 years (95% CI 1.6-3.3 years), and the median follow-up time in the good outcome group was 7.4 years (95% CI 6.1-8.2 years).

Dissociated fresh stage II primary melanoma tissue was obtained from 9 patients for flow cytometry analysis, although 3 samples had less than 100 T cells and were excluded from further analysis, leaving 6 samples. Due to the small size of this cohort and prospective manner of collection, the follow-up data are not reported in this study.

### Detailed analysis of CD8+ T cell populations in dissociated primary melanoma tissue

**Figure 1:**
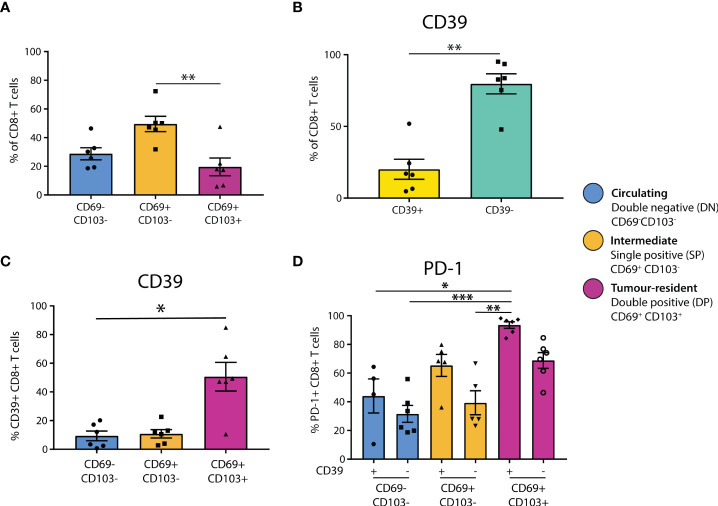
CD39+ tumor-resident CD8+ T cells are enriched for PD-1 in primary melanoma. **(A)** Flow cytometry was used to determine the residency status of CD8+ T cells in primary melanoma tissue dissociates (n = 6). Statistical differences were calculated using a non-parametric Kruskal-Wallis test **(B)** CD8+ T cell expression of CD39 was quantified. **(C)** CD39 expression was quantified in CD8+ T cell populations delineated by residency. **(D)** PD-1 expression was assessed by CD39 expression and tumor residency in CD8+ T cells. *p<0.05; **p<0.01, ***p<0.001.

To explore the T-cell phenotypes present in primary melanomas, six dissociated primary melanomas were stained for markers to investigate tumor residency and immune checkpoint receptor expression of CD8+ T cell subsets (**Figure 1**, **Supplementary Figure 1**). Flow cytometry analysis found CD8+ and CD4+ T cells at similar proportions after gating on total lymphocytes (CD4 mean = 38.92%, CD8 mean = 44.28%) (**Supplementary Figure 1B**).

To investigate tumor residency, in accordance with previous literature (13), CD8+ T cells were classified based on their expression of CD69 and CD103 as either double negative (DN, CD69-CD103-, ‘circulating’), single positive (SP, CD69+CD103-, ‘intermediate’), or double positive (DP, CD69+CD103+, ‘tumor-resident’, **Supplementary Figure 1A**). We found that the SP CD69+CD103- cells formed the largest group of CD8+ T cells in primary melanoma (DN mean = 28.78%, SP mean = 49.53%, DP mean = 19.64%), with the proportion of SP being significantly higher than that of DP (p=0.0088, **Figure 1A**). Characterization of CD4+ T cell residency was based on CD69+ expression (30, 31), finding that the majority of CD4+ T cells in primary melanoma are not tumor-resident (CD69+ = 26.5%, CD69- = 72.27%, p = 0.0022, **Supplementary Figure 1C**).

We then investigated CD39 expression in these CD8+ T populations, finding that the majority of CD8+ T cells in primary melanomas were CD39- (CD39+ mean = 20.11%, CD39- mean = 79.7%, **Figure 1B**). However, CD39 expression was then quantified separately within each CD8+ T cell residency group, finding that CD39 was expressed by the majority of DP tumor-resident CD8+ T cells (DN mean = 9.352%, SP mean = 10.78%, DP mean = 50.62%), with the CD39+ proportion being significantly higher in DP compared to DN (p = 0.0305, **Figure 1C**).

We then assessed PD-1 positivity based on CD39 expression and tumor residency. PD-1 expression was generally higher in CD39+ populations, and was highest in the CD39+ DP population, being almost ubiquitous in this group (CD39+ DN mean = 44.08%, CD39- DN mean = 31.57%, CD39+ SP mean = 65.4%, CD39- SP mean = 39.32%, CD39+ DP mean = 93.50%, CD39- DP mean = 68.83%, **Figure 1D**).

### Characterization of immune cell populations in primary melanoma tissue finds P2 (CD39+CD103+PD-1-) CD8+ T cells most closely associated with good outcomes

**Figure 2:**
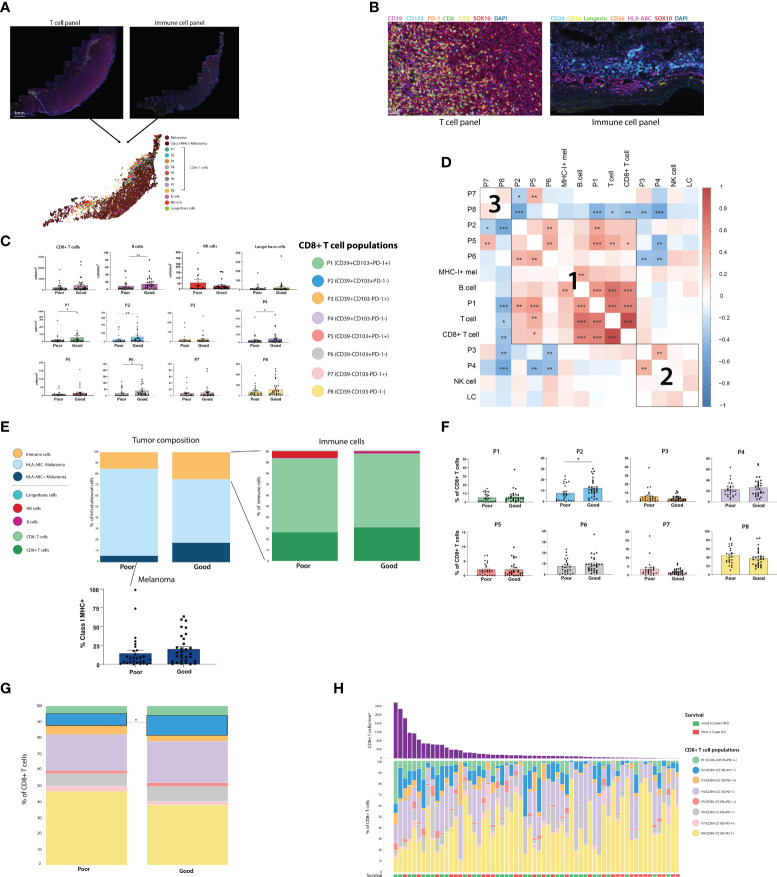
Assessing the composition of the primary melanoma intratumoral immune infiltrate finds CD39+CD103+PD-1- (P2) CD8+ T cells and B cells associated with improved outcome. **(A)** Two sections of primary melanoma FFPE samples were separately stained for CD8+ T cells and immune cells using Opal mIHC. Cells from separate sections were aligned to create a single spatial plot for each tumor. (n=64) **(B)** 20X resolution images of sections stained for T cell panel (left) and immune cell panel (right). **(C)** All immune cell populations were quantified per mm^2^ of tumor. Statistical differences were calculated using a Mann-Whitney unpaired non-parametric test. **(D)** Correlation plot of all immune cell populations, including % of each CD8+ T cell population, % Class I MHC positivity, and cells/mm^2^ of T cells, CD8+ T cells, B cells, NK cells and Langerhans cells (LC). **(E)** Tumor composition, intratumoral immune population composition, and % of Class I MHC+ melanoma were calculated. **(F)** Each of the 8 CD8+ T cell populations were quantified as a % of the total intratumoral CD8+ T cell compartment. **(G)** Composition of the intratumoral CD8+ T cell compartment overall was compared based on patient outcome. **(H)** Composition of the intratumoral CD8+ T cell compartment for individual patients. *p<0.05; **p<0.01, ***p<0.001.

To characterize these CD8+ T cell populations in a larger cohort, and investigate their spatial distribution and associations with outcome, we stained FFPE primary melanoma tissue from 66 patients with available clinical and follow-up data using mIHC. These populations were defined based on the data above as; P1 (CD39+CD103+PD-1+), P2 (CD39+CD103+PD-1-), P3 (CD39+ CD103-PD-1+), P4 (CD39+CD103-PD-1-), P5 (CD39- CD103+PD-1+), P6 (CD39-CD103+PD-1-), P7 (CD39- CD103-PD-1+) and P8 (CD39- CD103-PD-1-). Additionally, to assess CD8+ T cells in a broader immunological context, we also stained for B cells, NK cells, Langerhans cells and Class I MHC expression in a separate mIHC panel (**Figures 2A, B**).

Immune cells were quantified per mm^2^ in both the tumor (**Figure 2C**) and stromal regions (**Supplementary Figure 3**), and cell densities were compared between outcome groups. Total intratumoral CD8+ T cell, NK cell and Langerhans cell densities were not significantly different between Good and Poor outcome patients, while B cells were increased in patients with good outcomes. However, increased intratumoral densities of P1 (CD39+CD103+PD-1+), P2 (CD39+CD103+PD-1-), P4 (CD39+CD103-PD-1-) and P6 (CD39-CD103+PD-1-) were associated with Good outcomes. Correlation analysis between intratumoral immune cells identified 3 distinct clusters of immune cells which were more likely to be present within the same tumors. Firstly, cluster 1 consisted of CD103+ CD8+ T cell populations, with P1, P2, P5 and P6 frequently correlating together, along with B-cell and MHC-I+ tumor proportions. CD39+ CD103- populations P3 and P4 correlated with NK cells and Langerhans cells in cluster 2, and cluster 3 consisted solely of the CD39-CD103- CD8+ populations P7 and P8 (**Figure 2D**).

T cell fraction, defined as the number of T cells as a percentage of total nucleated cells from prior work (11), was not significantly different between outcome groups in this cohort (**Supplementary Figure 2B**). Class I MHC expression by melanoma cells was increased in patients with good outcomes, but not significantly so (Good mean = 20.07%, Poor mean = 14.5%, p = 0.0862, **Figure 2E**). Assessing CD8+ T cell phenotypes as a proportion of total CD8+ T cells (**Figures 2F–H**) found that P2 was higher in good outcome patients, and was the only population significantly associated with outcome (P2 Poor mean = 5.413%, P2 Good mean = 10.51%, p = 0.0136). Similar trends were observed in the stroma, with P2 being significantly increased in good outcome patients in both the cells/mm^2^ and percentage measures (**Supplementary Figures 3B–D**). Tertiary lymphoid structures (TLS), as defined by pathologist assessment, were not observed in any stromal tissue.

### B cells and CD103+PD-1- CD8+ T cells localize within close proximity of melanoma cells in patients with good outcomes

**Figure 3:**
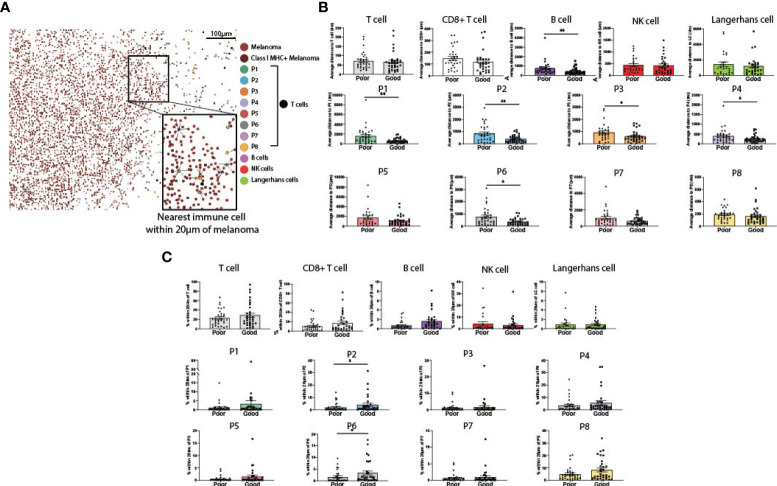
Proximity of melanoma to immune cell populations. **(A)** Cells from both panels were aligned and plotted onto a single spatial plot. Distances from melanoma to the nearest cell of each immune phenotype were calculated. **(B)** Average distance from melanoma to each immune phenotype was compared based on patient outcome. **(C)** % of melanoma cells within 20µm of each immune phenotype was compared based on patient outcome. Statistical differences were calculated using a Mann-Whitney unpaired non-parametric test.

Spatial analysis was performed on all immune cell types to determine their proximity to melanoma cells (**Figure 3A**). We first measured the average distance from a melanoma cell to the nearest immune cell (**Figure 3B**). While the proximity of total T cells and CD8+ T cells overall to melanoma was not associated with patient outcome, CD39+ CD8+ T cell phenotypes (P1-P4), P6 (CD39-CD103+PD-1-) and B cells were significantly closer to melanoma cells in patients with good outcomes (**Figure 3B**). We then measured proximity based on a 20µm cutoff (25), quantifying the percentage of melanoma cells within 20µm of each immune phenotype. This analysis found that P2 and P6 were within significantly closer proximity of melanoma cells in patients with good outcomes (**Figure 3C**).

### Analysis of immune cell localization at tumor margins find marginal B cells associated with good outcomes, and P1 and P2 at the infiltrating tumor edge

**Figure 4:**
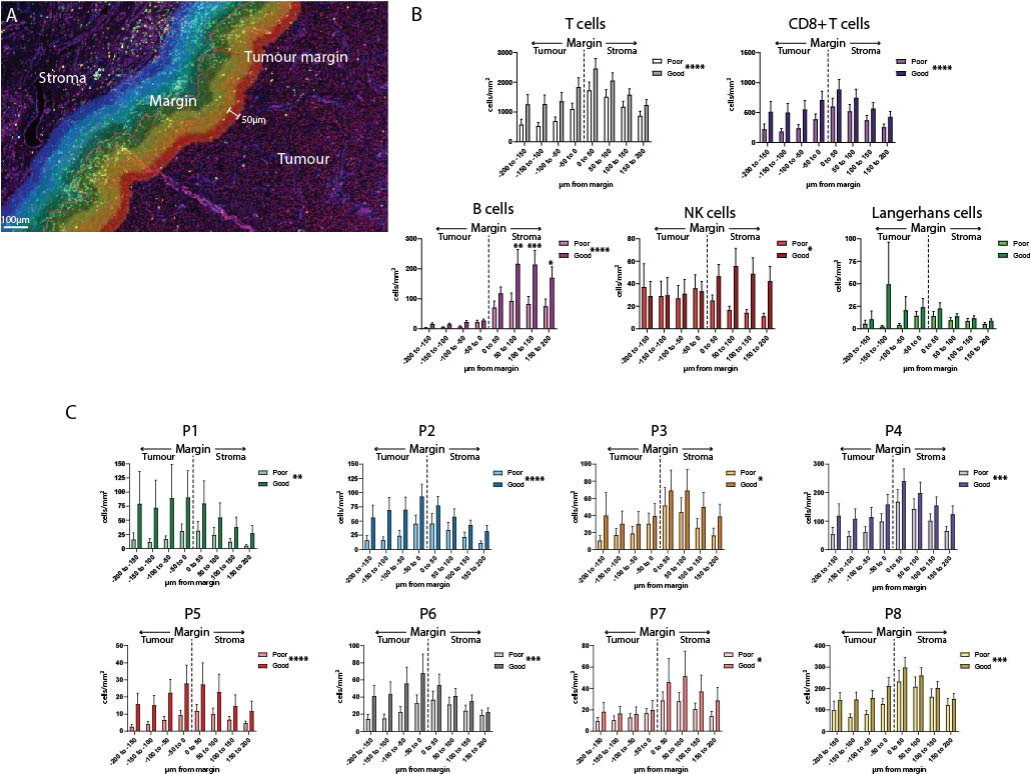
Analysis of the tumor margin finds increased B cells in the stroma. **(A)** A 400µm region surrounding the tumor margin was subdivided into 8 50um-diameter bands extending into the tumor and stroma. **(B)** Immune cells were quantified per mm^2^ in each band within the margin region and compared between outcome groups. **(C)** CD8+ T cell subsets were quantified per mm^2^ within each band of the margin region and compared between outcome groups. Overall significance is shown in the legend, significance per margin region is shown on the graph. Statistical differences were calculated using a 2-way ANOVA. *p<0.05; **p<0.01, ***p<0.001, ****p<0.0001.

While the previous spatial analysis investigated cells across the entire primary melanoma tissue, we wanted to look at cell interactions at the tumor/stromal interface or margin. We defined the marginal region as a 400µm-wide band, extending 200µm into the stroma and 200µm into the tumor as defined by the quantitative pathology software (**Figure 4A**). This region was further subdivided into 50µm bands, and immune cells were quantified separately per mm^2^ in each band (**Figure 4A**, **Supplementary Figure 4**). Analysis of each margin region found that B cells, NK cells, T cells and CD8+ T cells overall, as well as P3, P4, P7 and P8 individually, were more likely to localize in the stromal side of the margin (**Supplementary Data**). Notably, P5 and P6 localized close to the margin itself; and the only populations which were more likely to be found within the tumor-infiltrating face of the margin were P1 and P2 (**Supplementary Figure 4B**, **Supplementary Data**).

Differences in the composition of the tumor margin between outcome groups were compared overall, and individually within each 50µm margin region. Overall, significant differences were observed in all immune cell populations except for Langerhans cells (**Figures 4B, C**). Uniquely among all other immune cells, B cell levels in the stromal regions were significantly increased in patients with Good outcomes versus Poor outcomes (B cell 50-100µm p = 0.0008, 100-150µm p = 0.0021, 150-200µm p = 0.0412, **Figure 4B**).

### Analysis of CD8+ T-cell subsets along with clinicopathologic features to identify recurrence outcomes

**Figure 5:**
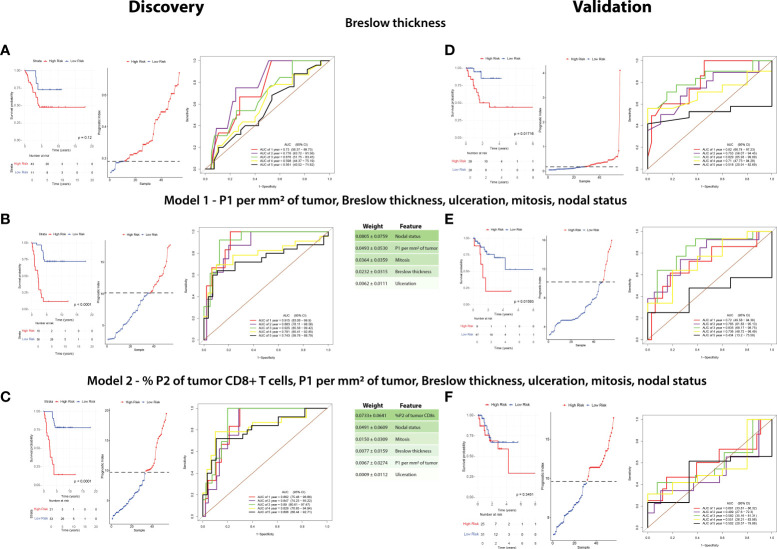
A combination of clinicopathologic characteristics with P1 CD8+ T cells is more predictive of patient outcome than Breslow thickness alone. A random survival forest model with exhaustive feature selection was fitted to identify the most predictive variables. RFS in the discovery cohort and validation cohorts were compared based on the three following factors: Breslow thickness **(A–D)**, an MVA including P1 per mm^2^ of tumor, Breslow thickness, ulceration, mitoses, and nodal status (Model 1, **B–E**), and an MVA including %P2 of tumor CD8+ T cells, P1 per mm^2^ of tumor, Breslow thickness, ulceration, mitoses, and nodal status (Model 2, **C–F**). Kaplan-Meier curves (left) were plotted for each model. High risk and low risk groups were determined using cutoffs which maximized the sensitivity and specificity of the ROC analysis. Prognostic index (PI) cutoff values for high risk were ≥0.18 in Breslow thickness, ≥9.810 (equivalent to ≥2.25mm Breslow thickness), ≥10.102 in Model 1, and ≥9.469 in Model 2, with low-risk values being below these cutoffs. Statistical differences were calculated using a log-rank test. A prognostic index plot shows the cutoff value, indicated by a dotted line, between high- and low-risk patients (middle). ROC curve analysis was reported at 1,2,3,4, and 5 years after diagnosis (right).

We then sought to determine the interaction between the CD8+ T-cell populations, clinicopathologic variables and disease recurrence. Therefore, we performed machine learning modelling to explore the contributions of CD8 + T-cell populations combined with all clinicopathologic variables (**Table 2**) to determine 5-year recurrence outcomes. However, given the discovery cohort in this study was matched per Breslow thickness groups per the recurrence outcomes, and given its clear significance, we explored Breslow thickness for an optimized cutoff value to identify its utility in this cohort. We found that a thickness of 2.25mm stratified patients by outcome, albeit not significantly (p = 0.12) with the highest accuracy of recurrence classification at 2 years (AUC = 77.6%, 95% CI = 63.72-91.56, **Figure 5A**).

A random survival forest model with exhaustive feature selection which examined combinations of CD8+ T cell proportions and clinicopathologic features was conducted. After AUC was calculated at all timepoints (death at years 1-5), we identified the combination with the highest time-dependent AUC to generate a model predictive of outcome. The model with the highest AUC included intratumoral P1/mm^2^, Breslow thickness, nodal status, mitotic rate and ulceration (Model 1, **Figure 5B**), with nodal status being the most strongly weighted feature. ROC analysis on the discovery cohort based on this model significantly stratified patients in the Kaplan-Meier RFS analysis [p <0.0001, 3-year AUC = 92.5% (95% CI = 85.58-99.42)].

We then identified the best performing model containing P2 proportions, as our above IHC analysis and univariate analysis found P2 was the most significantly different feature in the discovery cohort (Model 2, **Figure 5C**, **Table 2**). Interestingly, Kaplan-Meier analysis found that Model 2 was approximately as accurate as Model 1, however the peak AUC was lower [p<0.0001, 3-year AUC = 89% (95% CI = 80.61-97.47)]. However, in this model, % P2 of tumor CD8+ T cells was the most strongly weighted feature (0.0733 ± 0.0641) and the 4- and 5-year AUCs were higher than in Model 1 [4-year AUC = 82.9% (95% CI = 70.85-94.94), 5-year AUC = 80.6% (95% CI = 68.44-92.71), **Figure 5C**].

These models were applied to an independent validation cohort of primary melanoma samples from 80 patients. Within this cohort, 25 (31.25%) patients developed recurrence. The validation cohort was more frequently ulcerated (p = 0.0366) and had reduced Breslow thickness (p = 0.000874) compared to the discovery cohort, with a median time from primary melanoma diagnosis to recurrence of 1 year (95% CI = 0-2.11 years), while the median follow-up time for patients who remained recurrence-free was 1.67 years (95% CI = 1.25-2.10 years). As a reduced number of patients in the validation cohort had >3 years of follow-up, the accuracy of our analysis beyond 3 years was reduced. Furthermore, all CD8+ T cell populations assessed except for P4 were significantly reduced in the validation cohort (**Table 3**).

**Table 3:** Comparison of patient characteristics in discovery and validation cohorts.

Characteristics	Discovery (n = 54)	Validation (n = 56)	*p-value*
**Age**			
**Median (Range)**	67 (31-92)	70 (31-88)	0.8977
**Gender**			
**Female**	19 (35.2%)	19 (33.9%)	1.000
**Male**	35 (64.8%)	37 (66.1%)	
**Stage**			
**II (IIA/IIB/IIC)**	33 (61.1%)	42 (75.0%)	0.1742
**III (IIIA/IIIB/IIIC)**	21 (38.9%)	14 (25.0%)	
**Ulceration**			
**NO**	24 (44.4%)	37 (66.1%)	**0.0366**
**YES**	30 (55.60%)	19 (33.9%)	
**Nodal Status**			
0	33 (61.1%)	43 (76.8%)	0.1159
1	21 (38.9%)	13 (23.2%)	
**Breslow**			
**Median (Range)**	3.50 (1.35-9.0)	2.20 (0.6-50)	**0.000874**
**P1 mm2 tumor**			
**Median (Range)**	4.45 (0.0-981.26)	0.76 (0.0-192.41)	**0.009908**
**P2 mm2 tumor**			
**Median (Range)**	10.42 (0.14-622.31)	2.96 (0-307.44)	**0.01510**
**P4 mm2 tumor**			
**Median (Range)**	28.17 (1.68-696.15)	17.43 (0-1183.06)	0.08236
**P6 mm2 tumor**			
**Median (Range)**	9.18 (0-237.34)	4.94 (0.0 - 110.85)	**0.01921**
**% P2 tumor**			
**Median (Range)**	8.47 (0.01-30.23)	3.56 (0-65.85)	**0.01779**
**% P2 stroma**			
**Median (Range)**	6.12 (0.52-22.31)	0.77 (0.0-60.00)	**0.0002269**

Bold values indicate significant values.

As a benchmark, we performed a Kaplan-Meier analysis for Breslow thickness in the validation cohort which found similar results to that of the discovery cohort (p = 0.01716, 3-year AUC = 82.9% (95% CI = 63.93-99.89), **Figure 5D**). We then applied Model 1 to this cohort which significantly stratified patients based on Kaplan-Meier analysis using RFS as an endpoint (p = 0.001593, 3-year AUC = 83.5% (95% CI = 68.17-98.75%), **Figure 5E**). However, Model 2 was poorly validated in this cohort, with Kaplan-Meier analysis unable to stratify patients by risk (p = 0.3451, 1-year AUC = 60.1% (95% CI = 33.81-86.32%), **Figure 5F**).

## Discussion

The presence and distribution of tumor-infiltrating lymphocytes (TILs) as determined by histopathological assessment has been associated with the outcomes of primary melanoma patients for some time (7, 8). However, these features are not included in the AJCC staging system of primary melanoma patients. While investigations of the metastatic melanoma tumor immune microenvironment have yielded multiple potential biomarkers of response to immunotherapy (12), the immune landscape of primary melanoma remains poorly understood and immune biomarkers of patient outcome are lacking. Therefore this study sought to investigate the tumor microenvironment of primary melanoma and to determine if quantitative pathology and immune phenotyping could be additive to clinicopathologic features in identifying patients at a high risk of disease recurrence.

Through analysis of the primary melanoma tumor microenvironment focusing on CD8+ T cell phenotypes, we identified that high levels of CD39+CD103+PD-1- CD8+ T cells (P2) were associated with improved outcomes for primary melanoma patients in univariate analysis. Spatial analysis revealed that the localization of these immune populations in close proximity of the tumor were also closely associated with reduced rates of melanoma recurrence. P2 T cells were also within closer proximity to melanoma cells in patients with no recurrence, and alongside P1 (CD39+CD103+PD-1+) it was more likely to localize at the infiltrating face of the tumor margin. Based on its phenotype, P2 is tumor-resident (CD103+) and potentially specific for tumor antigens (CD39+) (14, 15). As such, it is likely to have high tumor reactivity and is a strong candidate for PD-1 upregulation, subsequently converting to the P1 phenotype (CD39+CD103+PD-1+). Of all the immune phenotypes in this study, only P1 and P2 were increased at the infiltrating front of the tumor margin, raising the possibility that these phenotypes are important for tumor control. Considering that high P1 proportions have been associated with recurrence-free survival in adjuvant anti-PD-1-treated stage III melanoma patients, increased P2 proportions in primary melanoma could possibly lead to improved responses to immunotherapy (28). Aside from P1, CD8+ T cell populations associated with improved outcomes in this study were PD-1 negative. This provides some support for the notion that a lack of PD-1 expression by CD8+ T cells could be beneficial to primary melanoma patient outcomes. In light of ongoing clinical trials of adjuvant anti-PD-1 therapy in stage II melanoma and the recent approval by the FDA for adjuvant pembrolizumab for stage II melanoma (5), investigating these CD8+ T cell populations within the context of primary melanoma immunotherapy is of great interest.

Our findings regarding B cells in this study were intriguing. We found B cells significantly associated with improved patient outcomes in three different contexts within the tumor microenvironment: increased intratumoral B cells, increased proximity of melanoma cells to B cells, and increased B cells closer to the stroma in the tumor margin region. These results were somewhat unexpected – although B cell levels were much higher in the stroma than the tumor, stromal B cell levels were not associated with patient outcome. Furthermore, intratumoral B cells were increased in patients with good outcomes, as was B cell proximity to tumors – despite the fact that the B cell humoral response, considered one of its most potent anti-tumor functions, is not dependent on contact with tumor cells (32, 33). Further complicating this, findings from previous studies of B cells in primary melanoma are conflicting and do not compare intratumoral and stromal B cells (19, 34–38). In other cancers and in immunotherapy-treated metastatic melanoma stromal B cells have been investigated as a part of TLSs (39). The presence of B cells in TLSs has been associated with response to immunotherapy (40–42). These studies also suggest that anti-tumor B cells act in tandem with other immune cells, and identify a CD19+CD27+IgD- B cell cluster enriched in responders. While we did not identify TLSs in our study, we did observe multiple lymphoid aggregates within the stromal regions of the tumor margin. As these lymphoid aggregates did not form the B cell follicle or T cell zone structures which define TLSs they may have been an early form of TLS (43). Since we found that B cells were significantly increased in the stromal region of the tumor margin, it is possible that these lymphoid aggregates or early TLSs contribute favorably to patient outcome (44). The exact anti-tumor function of B cells in our study is unclear. As well as producing anti-tumor antibodies able to facilitate tumor killing through antibody-dependent cell cytotoxicity (ADCC), B cells are also able to present antigens to CD4^+^ T cells (45). Indeed, we observe a high proportion of intratumoral and stromal CD8- T cells in our study. Although these cells could not be phenotyped in this investigation, their presence could be associated with the high B cell levels observed. Regulatory B cells (Bregs) are also of B cell lineage and act to suppress anti-tumor immunity. However, due to our finding that intratumoral B cells were increased in patient with good outcomes, it is unlikely that a significant proportion of B cells identified in this study were of the Breg phenotype. As our validation cohort was not stained for B cells we were unable to explore their prognostic potential in this study. Future investigations of B cells in primary melanoma should validate B cell associations with patient outcome, and additionally focus on B cell function and provide a more in-depth characterization of B cell phenotype.

Our cohort design allowed for correlation between immune cell phenotypes and outcomes independently of clinicopathological characteristics. This was an attempt to account for the association between Breslow thickness and ulceration with immune infiltration (4) and to enable the identification of immunological features which were independently associated with outcome from routine clinicopathologic characteristics (10, 11). Recent studies find that combining clinicopathologic characteristics with quantitative analysis of immune features can generate more powerful predictive models than interpreting these features on their own (11, 20). However, matching of patient clinicopathologic characteristics between outcome groups in our study posed difficulties in creating a predictive model which incorporated both immune and clinicopathologic features; as did the differences in stage at diagnosis between the validation cohort (stage I-IV) compared to the discovery cohort which only contained stage II and III patients, and the shorter follow-up period measured in the validation cohort. Our analysis was also limited to mainly larger, thicker tumors, with only 4 tumors in the discovery cohort having a Breslow thickness <2mm (46). Additionally, while whole slide image analysis was used in the discovery cohort, only 1mm^2^ cores were analyzed in the validation cohort. The smaller size of the tissue samples in the validation cohort reduced the number of CD8+ T cells available for analysis, which could have contributed to our inability to validate the predictive capability of % P2 of CD8+ T cells in the tumor. Given the limitations of the validation cohort, the models generated in this study should serve as an exploratory analysis with the purpose of providing proof of principle for the utility of quantitative pathology and to identify immune cell populations of interest for larger scale validation.

Comprehensive analysis of the clinicopathologic and immune features identified Model 1, containing nodal status, P1 (CD39+CD103+PD-1+) T-cells per mm^2^, mitotic rate, Breslow thickness and presence of ulceration, weighted in that order, as the most accurate model to predict melanoma recurrence in patients with primary melanoma. Meanwhile, P2 was weighted most strongly in Model 2, which could contribute to both its success in stratifying patients in the discovery cohort and its inability to do so in the validation cohort. It is also possible that P2 has an improved ability to predict long-term survival, but not short-term survival. Given that follow-up time for patients in the validation cohort was greatly reduced, while all surviving patients in the discovery cohort had >5 years of follow-up, further validation of P2 as a biomarker of outcome in primary melanoma is needed. With further validation, P1 and P2 CD8+ T cells and B cells could act independently as biomarkers for recurrence in primary melanoma. Identifying patients with a high risk of recurrence could allow these patients to be given adjuvant immunotherapy immediately following primary melanoma excision (5), which could greatly reduce their risk of recurrence.

One notable omission from our TME analysis was the characterization of myeloid cells such as macrophages. Similar studies in primary and metastatic melanoma characterizing macrophages solely on the basis of CD68 positivity have produced conflicting findings, with one study associating macrophages – particularly HLA-DR- macrophages – with a poor prognosis (22), while others found macrophages in primary melanomas were associated with improved survival (23, 47). Although CD68 has been commonly used as a pan-macrophage marker (48, 49), it is now accepted that macrophages – especially tumor-associated macrophages (TAM) – exist on a vast phenotypic and functional gradient, from anti-tumor-polarized M1 to pro-tumor M2 macrophages (50–52). On this basis, the accurate characterization of human macrophages based on mIHC alone could not be performed in this study with available tissue sections. In addition to macrophages, innate lymphoid cells (ILCs) and other myeloid populations such as neutrophils and eosinophils are understudied and difficult to characterize, particularly in primary melanoma (3). Studies of mouse models of melanoma have recently found that eosinophils recruited by ILC2 are associated with improved outcomes, and that boosting ILC function and eosinophil recruitment using combined anti-PD-1 and IL-33 induce anti-melanoma immunity. However, these results have not yet been observed in humans and tumor-associated ILC2 and eosinophils are difficult to characterize (53). Furthermore, while CD8+ T cells were well phenotyped in this study, our mIHC panel was not able to accurately characterize CD4+ T cells. Recent studies have identified CD39 as a marker of tumor-antigen specificity in conventional CD4+ T cells (Tconv) and found that these cells can be reactivated by anti-PD-1 therapy (54). However, both CD39 and PD-1 are also expressed by regulatory CD4+ T cells (Tregs), and CD69, rather than CD103, is a marker of tumor residency in CD4+ T cells (55). As neither our mIHC or flow cytometry were able to distinguish CD4+ T cell subsets, we were unable to characterize CD4+ T cells adequately in this study. More high-plexed techniques, such as CODEX, IMC and spatial RNAseq, could also allow for more in-depth phenotyping of these phenotypically complex populations. Additionally, while this study only investigated immune cell-tumor spatial relationships, future studies could perform more multidimensional neighborhood analysis to examine the spatial distribution of different immune cell populations with each other (36).

Immunophenotyping primary melanoma patient tumors in this study identified two immune cell populations significantly associated with improved outcomes: B cells, and a CD39+CD103+PD-1- CD8+ T cell population (P2). The localization of these immune cell populations provides further insight into their potential functions within the primary melanoma tumour microenvironment and raises questions as to their anti-tumor functionality. Furthermore, given further validation these populations could potentially provide added accuracy to routine clinical staging and allow for rationalized treatment and surveillance decisions to be made at the point of primary melanoma diagnosis.

## Data availability statement

The original contributions presented in the study are included in the article/**Supplementary Material**. Further inquiries can be directed to the corresponding author.

## Ethics statement

This study was reviewed and approved by Sydney Local Health District Human Ethics Review Committee. The patients/participants provided their written informed consent to participate in this study.

## Author contributions

Study design – GHA, HL, ATT, UP, PMF, JSW. Sample and clinical data acquisition – ATT, RPMS, JFT, PMF, RAS. Pathology review – PMF. Experiments and data analysis – GHA, ATT, ALF. Statistical models and analysis – NAA. Data interpretation – GHA, HL, ATT, NAA, IPdS, UP, JSW. Writing – original draft – GHA. Writing – review and editing – all authors. Supervision – UP, GVL, PMF, RAS, JSW. Funding – JFT, RAS, GVL. All authors contributed to the article and approved the submitted version.

## Funding

This work was supported by a National Health and Medical Research Council of Australia (NHMRC) Program Grant (APP1093017) to JFT, GVL and RAS. GHA is supported by scholarships from the University of Sydney and the Janet Ferguson MIA PhD Scholarship. HL was supported by an APEX Foundation scholarship. PMF was supported by the Deborah and John McMurtrie Melanoma Pathology Fellowship from Melanoma Institute Australia. GVL and JSW are supported by NHMRC Fellowships. GVL is also supported by the University of Sydney Medical Foundation. RAS is supported by an NHMRC Practitioner Fellowship (APP1141295). Support from The Ainsworth Foundation, The CLEARbridge Foundation, Cameron Family and Lady Mary Fairfax Charitable Trust are also gratefully acknowledged.

## Acknowledgments

We thank the patients and families involved in this study for their contributions to our research. Technical assistance from the Sydney Cytometry facility and support from the MIA Biospecimen Bank Team is gratefully acknowledged. We are also grateful for continued support from colleagues at Melanoma Institute Australia, Royal Prince Alfred Hospital, and the Charles Perkins Centre, the University of Sydney. Sections of this work were presented at the 18th International Congress of the Society for Melanoma Research, October 28-31st 2021.

## Conflict of interest

JFT has received honoraria for advisory board participation from BMS Australia, MSD Australia, GSK and Provectus Inc, and travel and conference support from GSK, Provectus Inc and Novartis. GVL is consultant advisor for Aduro Biotech Inc, Amgen Inc, Array Biopharma inc, Boehringer Ingelheim International GmbH, Bristol-Myers Squibb, Evaxion Biotech A/S, Hexel AG, Highlight Therapeutics S.L., Merck Sharpe and Dohme, Novartis Pharma AG, OncoSec, Pierre Fabre, QBiotics Group Limited, Regeneron Pharmaceuticals Inc, Specialised Therapeutics Australia Pty Ltd. RAS has received fees for professional services from F. Hoffmann-La Roche Lt​d, Evaxion, Provectus Biopharmaceuticals Australia, Qbiotics, Novartis, Merck Sharp and Dohme, NeraCare, AMGEN Inc., Bristol-Myers Squibb, Myriad Genetics, GlaxoSmithKline.

The remaining authors declare that the research was conducted in the absence of any commercial or financial relationships that could be construed as a potential conflict of interest.

The handling editor HPS declared a past co-authorship with the author RAS.

## Publisher’s note

All claims expressed in this article are solely those of the authors and do not necessarily represent those of their affiliated organizations, or those of the publisher, the editors and the reviewers. Any product that may be evaluated in this article, or claim that may be made by its manufacturer, is not guaranteed or endorsed by the publisher.

## Supplementary material

Supplementary Table 1 Detailed mIHC staining conditions for T cell panel.

Supplementary Table 2 Detailed mIHC staining conditions for immune cell panel.

Supplementary Figure 1 Flow cytometry gating in primary melanoma dissociates **(A)** Gating strategy for CD4+ T cells and CD8+ T cell populations based on residency. **(B)** Composition of CD4+ and CD8+ T cells (n=7). **(C)** CD69 expression by CD4+ T cells (n=6). Statistical differences were calculated using a Mann-Whitney unpaired non-parametric analysis.

Supplementary Figure 2 Previous models of outcome in primary melanoma were not predictive in this study. T cell fraction of total nucleated cells was assessed in the discovery cohort.

Supplementary Figure 3 Composition of immune cells in stroma. **(A)** Composition of the stroma as a percentage of each immune cell population was calculated. **(B)** Immune cells in the stroma were quantified per mm^2^ of stroma and compared based on patient outcome. **(C)** Composition of CD8+ T cells in the stroma was compared based on patient outcome. **(D)** Each CD8+ T cell population was calculated as a % of total stromal CD8+ T cells and compared based on patient outcome. Statistical differences were calculated using a Mann-Whitney unpaired non-parametric test.

Supplementary Figure 4 Composition of immune cells at tumor margin. Immune cells **(A)** and CD8+ T cell populations **(B)** were quantified per mm^2^ of each 50µm diameter band at the tumor margin. Immune cell levels were compared between margin regions. Statistical differences were calculated using a Kruskal-Wallis unpaired non-parametric test and are shown in Supplementary Data.

Supplementary Data Sheet Statistical differences of immune cells per mm^2^ between margin regions in **Supplementary Figure 4**.
