# Supplementary material for: Detailed spatial immunophenotyping of primary melanomas reveals immune cell subpopulations associated with patient outcome
Source: Front Immunol. 2022 Aug 8;13:979993. doi: 10.3389/fimmu.2022.979993 (PMC9393646; doi:10.3389/fimmu.2022.979993)
Supplement: Supplementary Table 1 — Detailed mIHC staining conditions for T cell panel. [file Table_1.docx]

**Supplementary Table 1**: Detailed mIHC staining conditions for T cell panel

| Order | Antibody | Source | Clone | Manufacturer | Dilution | Diluent | AR pH | Detection Kit | Opal |
| --- | --- | --- | --- | --- | --- | --- | --- | --- | --- |
| 1 | PD-1 | Rabbit | EPR4877(2) | Abcam | 1:1500 | Antibody Diluent/Block (Akoya Biosciences) | 9 | Mach 3 Rabbit HRP-polymer (Biocare Medical) | 540 |
| 2 | CD103 | Rabbit | EPR4166(2) | Abcam | 1:1500 | Antibody Diluent/Block | 9 | Opal Polymer HRP Ms + Rb (Akoya Biosciences) | 620 |
| 3 | CD8 | Mouse | C8/144B | Dako | 1:1500 | Antibody Diluent/Block | 9 | Opal Polymer HRP Ms + Rb | 650 |
| 4 | CD3 | Rabbit | MRQ-39 | Cell Marque | 1:1500 | Antibody Diluent/Block | 9 | Opal Polymer HRP Ms + Rb | 570 |
| 5 | CD39 | Rabbit | EPR20627 | Abcam | 1:2000 | Antibody Diluent/Block | 9 | Opal Polymer HRP Ms + Rb | 520 |
| 6 | SOX10 | Mouse | BC34 | Biocare Medical | 1:200 | Da Vinci Green (Biocare Medical) | 9 | Opal Polymer HRP Ms + Rb | 690 |
