# Supplementary material for: Detailed spatial immunophenotyping of primary melanomas reveals immune cell subpopulations associated with patient outcome
Source: Front Immunol. 2022 Aug 8;13:979993. doi: 10.3389/fimmu.2022.979993 (PMC9393646; doi:10.3389/fimmu.2022.979993)
Supplement: Supplementary Table 2 — Detailed mIHC staining conditions for immune cell panel. [file Table_2.docx]

| Order | Antibody | Source | Clone | Manufacturer | Dilution | Diluent | AR pH | Detection Kit | Opal |
| --- | --- | --- | --- | --- | --- | --- | --- | --- | --- |
| 1 | Langerin | Mouse | 12D6 | Cell Marque | 1:400 | Antibody Diluent/Block (Akoya Biosciences) | 6 | Mach 3 Mouse HRP-polymer (Biocare Medical) | 650 |
| 2 | CD56 | Rabbit | MRQ-42 | Cell Marque | 1:500 | Antibody Diluent/Block | 6 | Opal Polymer HRP Ms + Rb (Akoya Biosciences) | 570 |
| 3 | CD1a | Rabbit | EP80 | Cell Marque | 1:4000 | Antibody Diluent/Block | 6 | Opal Polymer HRP Ms + Rb | 540 |
| 4 | CD20 | Mouse | L26 | Biocare Medical | 1:800 | Antibody Diluent/Block | 6 | Opal Polymer HRP Ms + Rb | 520 |
| 5 | SOX10 | Mouse | BC34 | Biocare Medical | 1:200 | Da Vinci Green (Biocare Medical) | 6 | Opal Polymer HRP Ms + Rb | 690 |
| 6 | HLA-ABC | Mouse | EMR8-5 | Abcam | 1:20000 | Antibody Diluent/Block | 6 | Opal Polymer HRP Ms + Rb | 620 |

**Supplementary Table 2**: Detailed mIHC staining conditions for immune cell panel
