# Supplementary figures and images for: Detailed spatial immunophenotyping of primary melanomas reveals immune cell subpopulations associated with patient outcome
Source: Front Immunol. 2022 Aug 8;13:979993. doi: 10.3389/fimmu.2022.979993 (PMC9393646; doi:10.3389/fimmu.2022.979993)

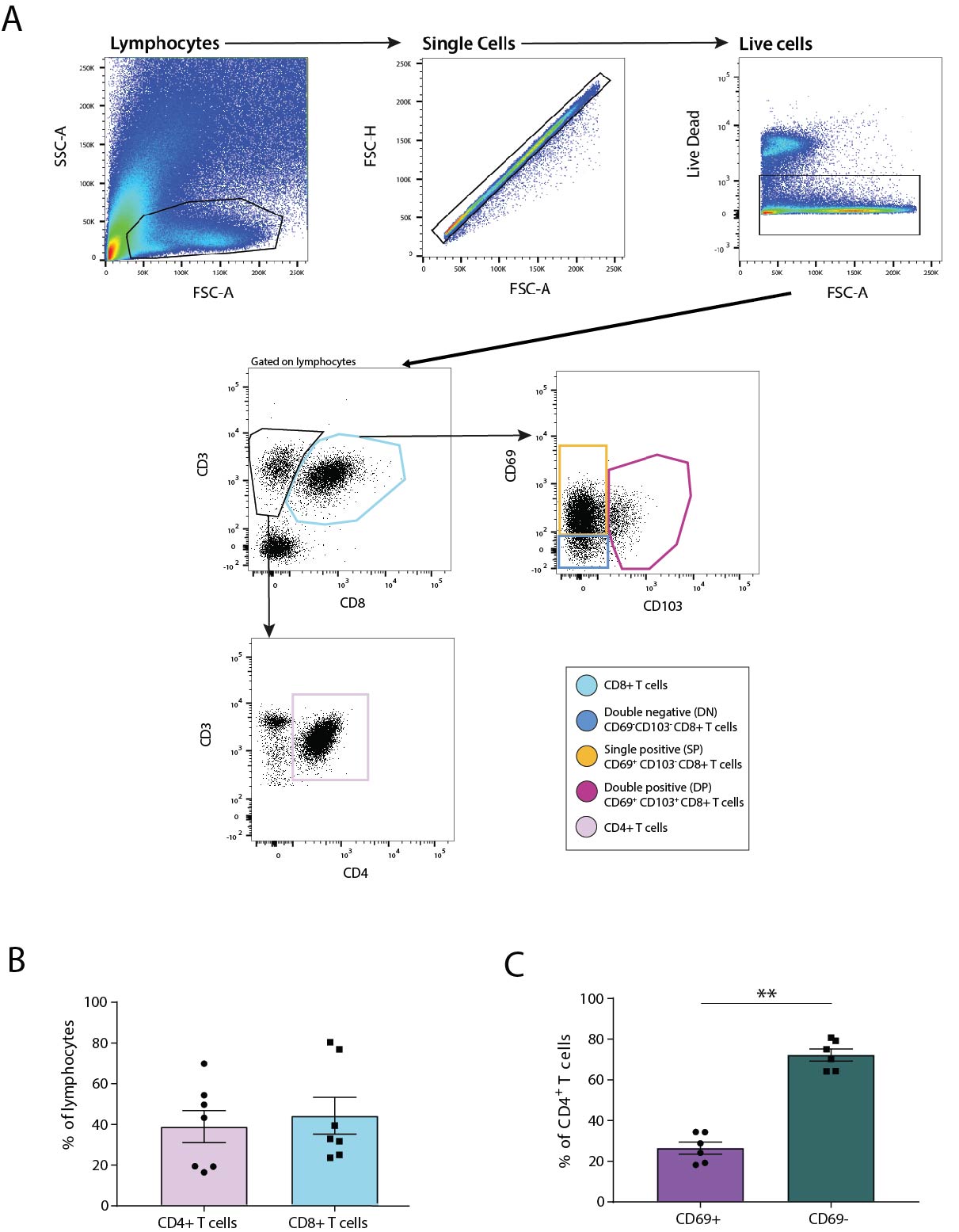

Supplement: Supplementary Figure 1 — Flow cytometry gating in primary melanoma dissociates (A) Gating strategy for CD4+ T cells and CD8+ T cell populations based on residency. (B) Composition of CD4+ and CD8+ T cells (n=7). (C) CD69 expression by CD4+ T cells (n=6). Statistical differences were calculated using a Mann-Whitney unpaired non-parametric analysis. [file Image_1.jpeg]

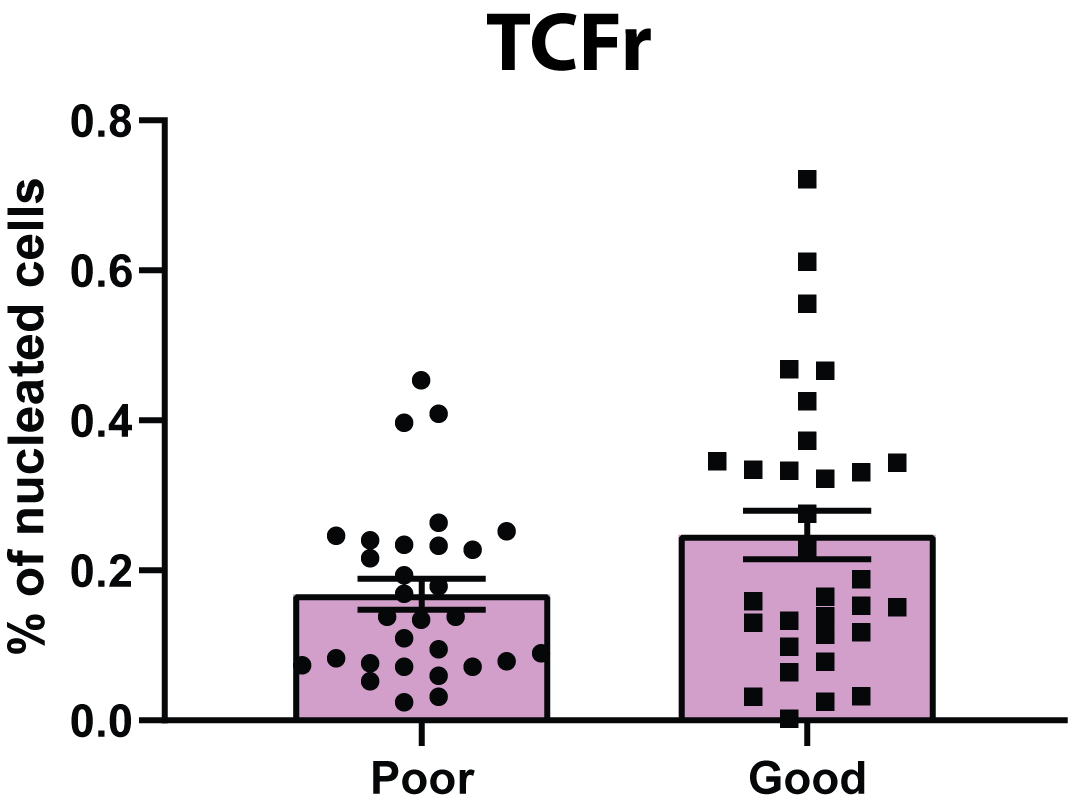

Supplement: Supplementary Figure 2 — Previous models of outcome in primary melanoma were not predictive in this study. T cell fraction of total nucleated cells was assessed in the discovery cohort. [file Image_2.tif]

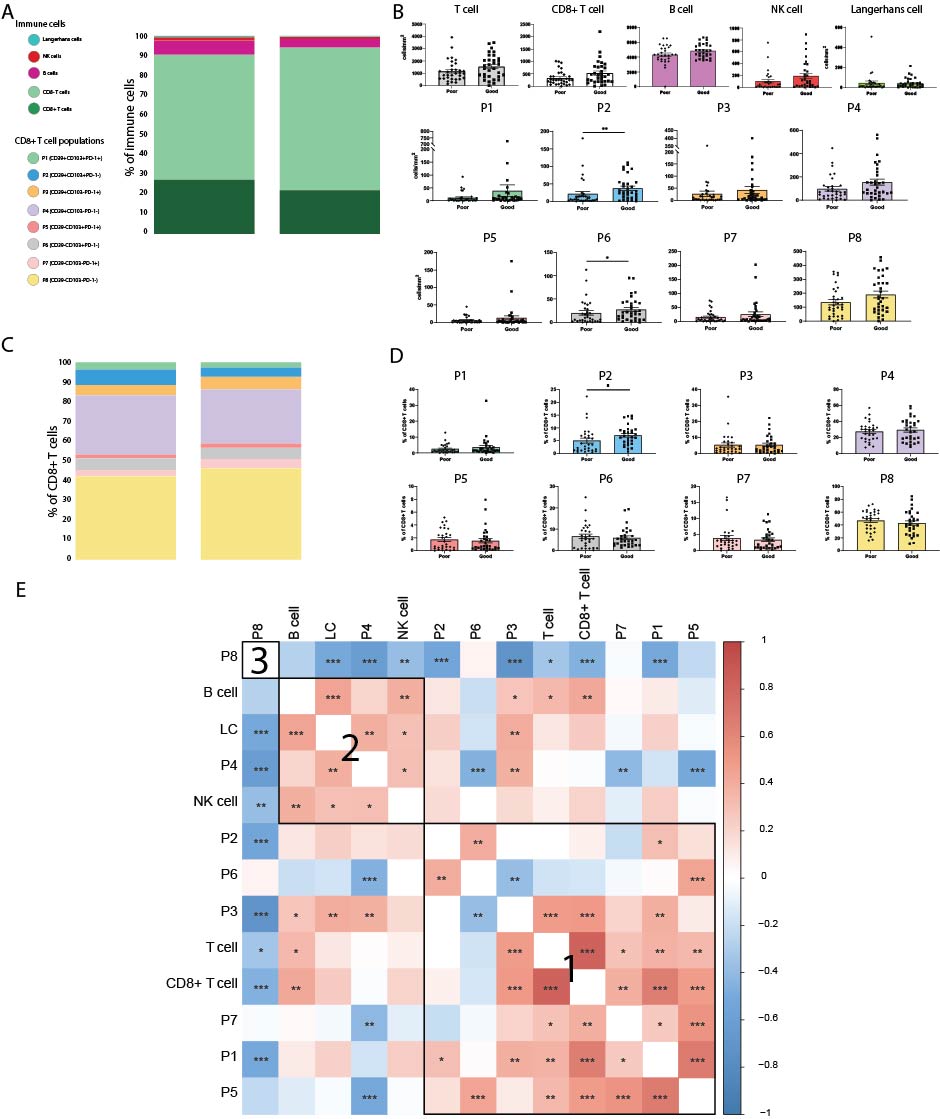

Supplement: Supplementary Figure 3 — Composition of immune cells in stroma. (A) Composition of the stroma as a percentage of each immune cell population was calculated. (B) Immune cells in the stroma were quantified per mm2 of stroma and compared based on patient outcome. (C) Composition of CD8+ T cells in the stroma was compared based on patient outcome. (D) Each CD8+ T cell population was calculated as a % of total stromal CD8+ T cells and compared based on patient outcome. Statistical differences were calculated using a Mann-Whitney unpaired non-parametric test. [file Image_3.jpeg]

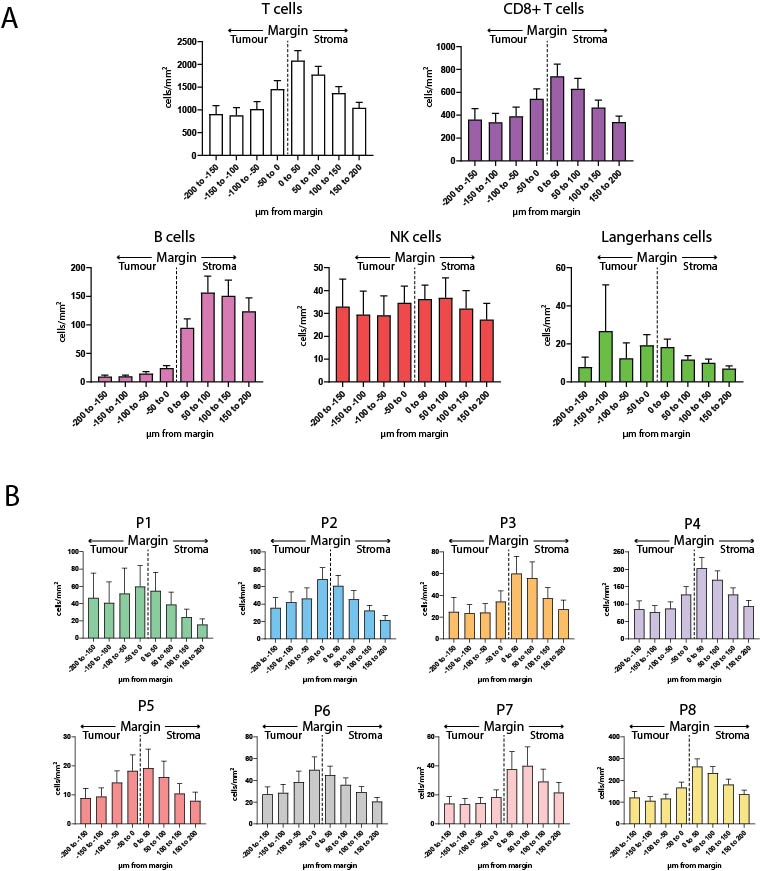

Supplement: Supplementary Figure 4 — Composition of immune cells at tumor margin. Immune cells (A) and CD8+ T cell populations (B) were quantified per mm2 of each 50µm diameter band at the tumor margin. Immune cell levels were compared between margin regions. Statistical differences were calculated using a Kruskal-Wallis unpaired non-parametric test and are shown in Supplementary Data. [file Image_4.jpeg]
